# Trends in Cervical Precancers Identified Through Population-Based Surveillance — Human Papillomavirus Vaccine Impact Monitoring Project, Five Sites, United States, 2008–2022

**DOI:** 10.15585/mmwr.mm7406a4

**Published:** 2025-02-27

**Authors:** Julia W. Gargano, Ruth Stefanos, Rebecca M. Dahl, Jessica L. Castilho, Erica A. Bostick, Linda M. Niccolai, Ina U. Park, Sheelah Blankenship, Monica M. Brackney, Kameny Chan, Emily L. Delikat, Sara Ehlers, Kimberly Gonzalez Barrera, RaeAnne Kurtz, James I. Meek, Erin Whitney, Marissa Vigar, Elizabeth R. Unger, Lauri E. Markowitz, HPV-IMPACT Working Group, HPV-IMPACT Working Group, Deborah Adeyemi, Anjola-Oluwa A. Ajayi, Nicole R. Andersen, Bradley Beauchamp, Sarah E. Clarke, Emilio DeBess, Kyle Higgins, Tiffanie M. Markus, Troy D. Querec, Michael Silverberg

**Affiliations:** ^1^Division of Viral Diseases, National Center for Immunization and Respiratory Diseases, CDC; ^2^Epidemic Intelligence Service, CDC; ^3^Division of Infectious Diseases, Vanderbilt University Medical Center, Nashville, Tennessee; ^4^Department of Health Policy, Vanderbilt University Medical Center, Nashville, Tennessee; ^5^University of Rochester School of Medicine and Dentistry, Rochester, New York; ^6^Connecticut Emerging Infections Program, Yale School of Public Health, New Haven, Connecticut; ^7^Department of Family and Community Medicine, University of California-San Francisco School of Medicine, San Francisco, California; ^8^Oregon Health Authority; ^9^California Emerging Infections Program, Richmond, California; ^10^Division of High-Consequence Pathogens and Pathology, National Center for Emerging and Zoonotic Infectious Diseases, CDC.; California Emerging Infections Program; Vanderbilt University Medical Center; Vanderbilt University Medical Center; Oregon Health Authority; Vanderbilt University Medical Center; Oregon Health Authority; Connecticut Emerging Infections Program; Vanderbilt University Medical Center; CDC; Kaiser Permanente Northern California.

**Summary**

**What is already known about this topic?**

Since 2006, when human papillomavirus (HPV) vaccine was first recommended in the United States to prevent cancers and other diseases caused by HPV, vaccination coverage has increased, and many young women vaccinated as children or adolescents have become age-eligible for cervical cancer screening. CDC monitors cervical precancer incidence through the Human Papillomavirus Vaccine Impact Monitoring Project.

**What is added by this report?**

During 2008–2022, cervical precancer incidence decreased 79% and higher-grade precancer incidence decreased 80% among screened women aged 20–24 years, the age group most likely to have been vaccinated.

**What are the implications for public health practice?**

Observed declines in cervical precancers are consistent with HPV vaccination impact and support Advisory Committee on Immunization Practices recommendations to vaccinate children against HPV at age 11–12 years with catch-up through age 26 years.

## Abstract

In 2006, human papillomavirus (HPV) vaccine was first recommended in the United States to prevent cancers and other diseases caused by HPV; vaccination coverage increased steadily through 2021, and increasing numbers of young women had received HPV vaccine as children or adolescents. Since 2008, CDC has monitored incidence of precancerous lesions (cervical intraepithelial neoplasia [CIN] grades 2–3 and adenocarcinoma in situ [AIS], collectively CIN2+), which are detected through cervical cancer screening and can be used as an intermediate outcome for monitoring vaccination impact, via the five-site Human Papillomavirus Vaccine Impact Monitoring Project. This analysis describes trends in incidence of CIN2+ and CIN3+ (i.e., CIN grade 3 and AIS) lesions during 2008–2022. Among women aged 20–24 years who were screened for cervical cancer, rates during 2008–2022 decreased for CIN2+ by 79%, and for CIN3+ by 80%. In the same period, CIN3+ rates among screened women aged 25–29 years decreased by 37%. These data are consistent with considerable impact of HPV vaccination for preventing cervical precancers among women in the age groups most likely to have been vaccinated, and support existing recommendations to vaccinate children at the routinely recommended ages as a cancer prevention measure.

## Introduction

Human papillomavirus (HPV) causes approximately 10,800 cervical cancers in the United States each year; cervical cancer is the most common HPV-attributable cancer among women (*1*). HPV-attributable cancers take many years to develop (median age at diagnosis = 50 years),* whereas screen-detected cervical precancers (cervical intraepithelial neoplasia [CIN] grades 2–3 and adenocarcinoma in situ [AIS], collectively CIN2+) can develop within a few years after infection (*2*). Screening with the Papanicolaou (Pap) test and treatment of precancerous abnormalities have been the mainstay of secondary prevention of cervical cancer for decades; screening with a test for high-risk HPV, usually as a co-test with a Pap test, has been increasingly used during the past decade (*2*,*3*). Recommended screening intervals have increased, from annually to every 3 years (cytology only) or every 5 years (incorporating an HPV test). In 2006, HPV vaccine was first recommended in the United States by CDC’s Advisory Committee on Immunization Practices to prevent cancers and other diseases caused by HPV^†^ (*2*). Routine vaccination was first recommended for girls and women; in 2011, boys and men were included in the vaccination program. Currently, routine vaccination is recommended for all children at age 11–12 years (may commence at age 9 years), with catch-up vaccination through age 26 years (*2*). Since 2019, shared clinical decision-making has been recommended for consideration of vaccination of adults aged 27–45 years. Two doses of HPV vaccine are recommended if the series is started at age <15 years; otherwise, 3 doses are recommended. Coverage with ≥1 HPV vaccine dose among adolescents aged 13–17 years steadily increased through 2021 then plateaued, with most recent coverage of 76.8% in 2023 (*4*). CDC has monitored CIN2+ incidence since 2008 via the Human Papillomavirus Vaccine Impact Monitoring Project (HPV-IMPACT) (*5*,*6*). This analysis describes trends in CIN2+ and CIN3+ (i.e., CIN grade 3 and AIS) incidence during 2008–2022.

## Methods

### Surveillance System

HPV-IMPACT has conducted population-based CIN2+ surveillance in five sites since 2008.^§^ Participating sites conduct active surveillance of all histopathology laboratories serving catchment area residents to identify histologically confirmed CIN2+ diagnoses.

### Populations and Screening Estimation

The number of women aged 20–64 years in the HPV-IMPACT catchment areas was obtained from U.S. Census Bureau data, stratified by 5-year age group.^¶^ The proportions of women screened (i.e., who had a Pap or HPV test in the preceding year), by 5-year age group, were estimated** using standardized data sources and methods for all sites^††^ (*5*,*6*). To account for variations in screening by insurance coverage, estimates are weighted averages of proportions screened among privately insured,^§§^ publicly insured,^¶¶^ and uninsured women.*** Annual age-specific numbers of women screened were estimated by multiplying age-specific proportions screened by age-specific populations of women.

### Statistical Analysis

For the years 2008–2022, annual age-specific CIN2+ and CIN3+ incidence and 95% CIs were calculated using the estimated number of women screened as the denominator to control for changes in screening frequency; incidence is still affected by changes in screening test sensitivity and vaccination coverage. Incidence calculations were performed using SAS (version 9.4; SAS Institute). Age-specific trend analyses were conducted separately for CIN2+ and CIN3+ using Joinpoint software (version 5.3.0.0; National Cancer Institute).^†††^ Overall trends for 2008–2022 were reported as average annual percent change (AAPC), and segment-specific trends were reported as annual percent change (APC).^§§§^ Because health care disruptions due to the COVID-19 pandemic might have resulted in deviations in incidence in 2020 from the underlying trend, data from 2020 were excluded from trend analyses.^¶¶¶^ This activity was reviewed by CDC, deemed not research, and was conducted consistent with applicable federal law and CDC policy.****

## Results

During 2008–2022, a total of 39,977 CIN2+ cases were reported; 13,027 (32.6%) were CIN3+. CIN2+ cases per 100,000 screened women decreased 11.0% annually for women aged 20–24 years; the incidence in 2022 was 79.5% lower than that in 2008 (Table) (Figure) (Supplementary Table, https://stacks.cdc.gov/view/cdc/176064). Among screened women aged 25–29 years, CIN2+ incidence increased 3.1% annually during 2008–2016, and decreased 4.3% annually during 2016–2022, but AAPC was stable. Among women aged 30–34 years and 35–39 years, CIN2+ incidence trended upward during 2008–2016 and then downward during 2016–2022; AAPCs for the entire period were positive but small. Among those in age groups 40–49 and 50–64 years, CIN2+ incidence increased significantly during 2008–2022.

**TABLE:** Average annual percent change and annual percent change for cervical precancers per 100,000 screened women* — Human Papillomavirus Vaccine Impact Monitoring Project,^†^ five sites, United States, 2008–2022

Age group, yrs	(95% CI)			
	CIN2+^§^ cases per 100,000 screened women		CIN3+^¶^ cases per 100,000 screened women	
	AAPC**	APC**	AAPC**	APC**
20–24	−11.0 (−12.8 to −10.2)^††^	2008–2012: −5.9 (−9.0 to 0.2) 2012–2022: −12.9 (−16.7 to −11.8)^††^	−10.2 (−13.0 to −9.0)^††^	2008–2013: −7.9 (−10.7 to −2.9)^††^ 2013–2018: −18.0 (−28.1 to −13.7)^††^ 2018–2022: −2.7 (−14.2 to 13.4)
25–29	−0.2 (−2.5 to 2.3)	2008–2016: 3.1 (1.1 to 16.7)^††^ 2016–2022: −4.3 (−14.6 to −0.7)^††^	−3.5 (−5.4 to −2.1)^††^	2008–2016: 0.7 (−1.2 to 4.4) 2016–2022: −8.8 (−16.2 to −5.3)^††^
30–34	2.4 (1.3 to 3.5)^††^	2008–2012: 2.6 (−6.5 to 7.7) 2012–2016: 12.3 (7.1 to 18.8)^††^ 2016–2022: −3.8 (−7.0 to −1.6)^††^	1.0 (−0.1 to 2.1)	2008–2012: 2.3 (−6.5 to 6.9) 2012–2016: 12.6 (7.4 to 18.8)^††^ 2016–2022: −6.8 (−10.0 to −4.4)^††^
35–39	3.0 (2.0 to 3.9)^††^	2008–2012: 3.0 (−4.4 to 7.4) 2012–2016: 12.1 (7.3 to 18.1)^††^ 2016–2022: −2.7 (−5.8 to −0.6)^††^	2.6 (0.1 to 6.0)^††^	2008–2017: 5.2 (2.1 to 29.3)^††^ 2017–2022: −2.0 (−13.7 to 3.9)
40–49	4.4 (2.2 to 7.5)^††^	2008–2017: 7.5 (5.5 to 26.6)^††^ 2017–2022: −0.8 (−11.9 to 4.0)	3.9 (2.0 to 5.8)^††^	2008–2022: 3.9 (2.0 to 5.8)^††^
50–64	5.2 (4.1 to 7.0)^††^	2008–2010: 1.9 (−6.2 to 13.8) 2010–2016: 12.8 (−4.7 to 21.9) 2016–2022: −0.7 (−4.7 to 4.6)	4.2 (2.1 to 7.3)^††^	2008–2017: 7.2 (5.0 to 27.5)^††^ 2017–2022: −0.9 (−11.6 to 4.2)

**Abbreviations:** AAPC = average annual percent change; APC = annual percent change; CIN = cervical intraepithelial neoplasia; HPV-IMPACT = Human Papillomavirus Vaccine Impact Monitoring Project.

* Denominators are the estimated number of screened women aged 20–24, 25–29, 30–34, 35–39, 40–49, or 50–64 years living in the catchment areas.

^†^ HPV-IMPACT sites are Alameda County, California; New Haven County, Connecticut; Monroe County, New York; Davidson County, Tennessee; and 28 zip codes in metropolitan Portland, Oregon.

^§^ CIN2+ includes grades 2 or worse and adenocarcinoma in situ.

^¶^ CIN3+ includes grade 3 and adenocarcinoma in situ.

** Data from 2020 were excluded from trend analyses. Analyses were also performed with 2020 data included. Most AAPCs were similar (within approximately 0.3) and did not change statistical significance. The CIN2+ AAPC among age group 50–64 years increased from 5.2 to 6.5, and the increase in CIN3+ among age group 35–39 years was similar but not statistically significant. There were also some changes in the years of identified joinpoints that would not change overall interpretations.

^††^ Denotes APC or AAPC is significantly different from zero at the alpha = 0.05 level.

**FIGURE:**
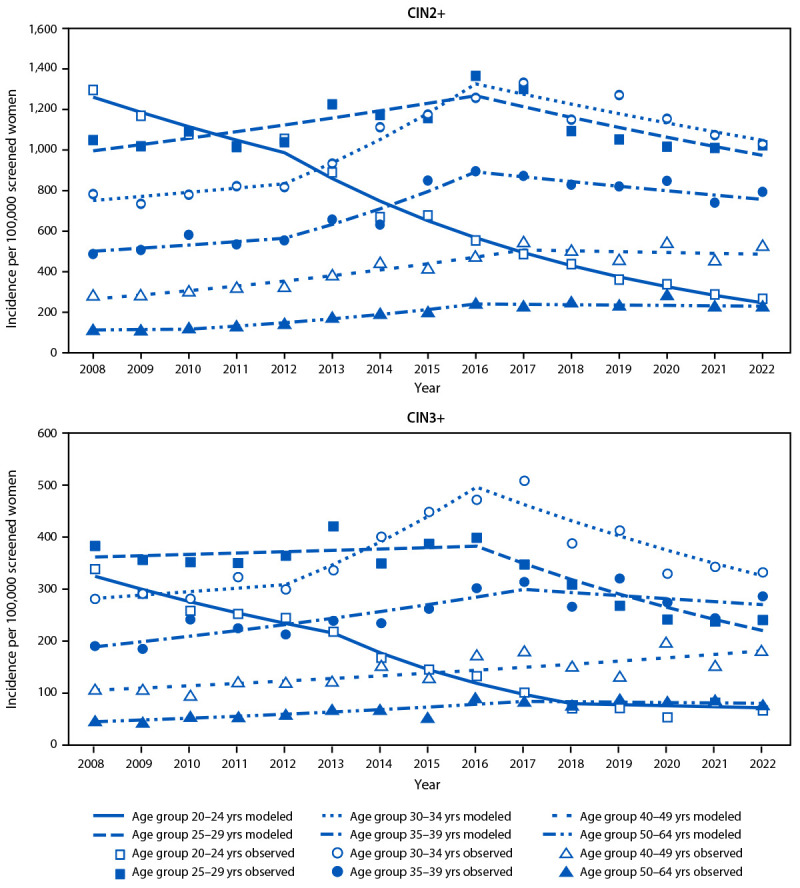
Incidence (cases per 100,000 screened women)* of cervical precancers^†^ — Human Papillomavirus Vaccine Impact Monitoring Project,^§^ five sites, United States, 2008–2022^¶^ **Abbreviations:** CIN = cervical intraepithelial neoplasia; HPV-IMPACT = Human Papillomavirus Vaccine Impact Monitoring Project. * Denominators are the estimated number of screened women aged 20–24, 25–29, 30–34, 35–39, 40–49, or 50–64 years living in the catchment areas. ^†^ CIN2+ includes grades 2 or worse and adenocarcinoma in situ; CIN3+ includes grade 3 and adenocarcinoma in situ. ^§^ HPV-IMPACT sites are Alameda County, California; New Haven County, Connecticut; Monroe County, New York; Davidson County, Tennessee; and 28 zip codes in metropolitan Portland, Oregon. ^¶^ Trend lines were modeled using Joinpoint software (version 5.3.0.0; National Cancer Institute). Data from 2020 were excluded from trend analyses.

Trends for CIN3+ were generally similar to those for CIN2+; among women aged 20–24 years, the incidence in 2022 was 80.3% lower than that in 2008. One notable difference for CIN3+ compared with CIN2+ was an overall decreasing trend among screened women aged 25–29 years (AAPC = −3.5%); the CIN3+ incidence in 2022 was 37.2% lower than that in 2008.

## Discussion

These data from HPV-IMPACT provide an updated view into the epidemiology of cervical precancers in the United States during an era of increasing HPV vaccination coverage among young women and changing cervical cancer screening practices among all age groups. Among women aged 20–24 years who were screened, CIN2+ incidence decreased 79% from 2008 to 2022, and CIN3+ incidence decreased 80%. Among screened women aged 25–29 years, CIN3+ incidence decreased 37%. The data are consistent with a considerable impact from the U.S. HPV vaccination program on cervical precancers, with the largest decreases in the youngest age group for which benefit of vaccination would first be observed.

Data from HPV-IMPACT previously indicated decreasing CIN2+ and CIN3+ incidence among women aged 18–20 and 21–24 years during 2008–2015 (*6*). As vaccinated women age into older age groups, declines in cervical precancers are expected. For example, before 2014, women aged 20–24 years could only have been vaccinated in the catch-up vaccination age range (13–26 years), whereas during 2018–2022, all women aged 20–24 years would have been eligible for vaccination at the routine age (11–12 years) in 2006. Vaccination at the routine age is more effective because vaccination is likely to occur before exposure to HPV through sexual contact. The declines in precancers mirror reported U.S. trends in vaccine-type HPV prevalence in self-collected cervicovaginal swabs, in which declines in quadrivalent HPV-type prevalence among adolescents and women aged 14–19 years were followed by declines among women aged 20–24 years (*2*). This report includes the first U.S. data showing significant decreases in cervical precancers in an older age group: CIN3+ incidence among screened women aged 25–29 years decreased compared with incidence during the beginning of the surveillance period. Most women aged 25–29 years in 2022 had been eligible for vaccination at age 11–12 years. The decrease in CIN3+ incidence before CIN2+ could be because CIN3+ (compared with CIN2+ lesions) are more frequently positive for HPV16 or HPV18, and therefore, a higher proportion are preventable by quadrivalent HPV vaccination (*5*,*7*).

HPV-IMPACT previously reported increasing trends in precancer incidence among women in age groups 25–39 years during 2008–2015 (*6*). Increases were attributed to longer screening intervals (all ages) and increasing use of HPV testing (age ≥30 years), which are more sensitive for detection of CIN2+ than Pap tests; HPV testing has increased during the surveillance period (*3*). Continued HPV-IMPACT surveillance indicated that increasing CIN2+ incidence among several age groups from 25 to 64 years reversed or leveled during 2016–2017. By 2022, incidence among women aged 25–29 years had decreased to near 2008 levels (CIN2+) or below (CIN3+). These findings are consistent with model predictions that changing from Pap to HPV testing would cause transient increases in detected precancer and cancer, followed by decreases (*8*). Although some women in age groups 30–34 years and older would have been vaccinated, less impact is expected among women in this age group at this time because they were only eligible for catch-up vaccination, at ages when many women are already sexually experienced and therefore likely to have been infected with HPV.

### Limitations

The findings in this report are subject to at least four limitations. First, numbers of women screened for cervical cancer were estimated using claims and survey data; inaccuracies could lead to under- or overestimates of precancer rates among screened women. Second, changes in screening and management guidelines and uncertainty in histologic classification could have affected CIN2+ and CIN3+ case detection. Third, data are limited to ecologic trends, and interpretations inferring relationships between vaccination and precancer incidence lack causal certainty; however, trend analyses such as these are routinely used to evaluate the impact of vaccination programs,^††††^ and no other plausible explanations for the decreases in precancers have been identified. Finally, because one site’s catchment area expanded and the overall program standardized its screening estimation methods, numbers are not directly comparable with those reported in previous publications from this project.

### Implications for Public Health Practice

These data are consistent with continuing impact of the U.S. HPV vaccination program on reducing cervical precancers (including CIN3+, the outcome most proximal to cervical cancer), and are consistent with both declines in vaccine-type HPV prevalence and early observations of reductions in cervical cancer among young women (*2*,*9*,*10*). The data also suggest that precancer incidence in age groups ≥25 years, which were previously observed to increase through 2015, have begun to decrease. HPV vaccination^§§§§^ and guidelines-based cervical cancer screening^¶¶¶¶^ are important tools for cervical cancer prevention.

## Footnotes

*
https://www.cdc.gov/cancer/hpv/diagnosis-by-age.html

†
https://www.cdc.gov/acip-recs/hcp/vaccine-specific/hpv.html

§ This report includes data submitted through August 2024. HPV-IMPACT sites are Alameda County, California; New Haven County, Connecticut; Monroe County, New York; Davidson County, Tennessee; and 28 zip codes in metropolitan Portland, Oregon. Originally, the California site included portions of Alameda County but was expanded retroactively to include the full county. Additional information about the surveillance system is available at https://www.cdc.gov/hpv-impact/about/index.html.

¶ For the California, Connecticut, New York, and Tennessee sites, 2022 annual U.S. Census Bureau county estimates were used. The Connecticut site transitioned from counties to planning regions in 2022, but HPV-IMPACT maintained the original catchment area for the period of this report; the 2021 county U.S. Census Bureau population was used as a proxy for the 2022 population. For the Oregon site, annual demographic projections based on U.S. Census Bureau data were used.

** Estimation is necessary because no national or catchment area–specific registries for cervical cancer screening exist in the United States, and surveillance of cervical cytology laboratories is outside the scope of HPV-IMPACT surveillance.

†† Methodology was similar to that used for the California site in previous reports, but with data sources standardized for all sites. For U.S. Census Bureau American Community Survey Public Use Microdata Areas corresponding to each catchment area, the proportion of women with public, private, or no insurance was obtained for the years 2008–2022 (https://www.census.gov/programs-surveys/acs/). Total proportion screened was a weighted average of the proportion screened for each of the three insurance categories.

§§ The age-specific proportions of privately insured women who were screened were estimated using claims data from the Merative MarketScan Commercial Database. The data were used to obtain claims for Pap or HPV testing at the Metropolitan Statistical Area level for each site, 2008–2022. Pap or HPV testing claims among persons who also had a claim for hysterectomy were excluded.

¶¶ The age-specific proportions of publicly insured women who were screened were estimated using claims data from the Merative MarketScan Multi-State Medicaid Database. The data were analyzed for Pap or HPV testing obtained nationally during 2008–2022. Pap or HPV testing claims among women who also had a claim for hysterectomy were excluded. For each site, these claims were adjusted based on the ratio between site-specific commercial claims and national commercial claims.

*** The age-specific proportions of uninsured women who were screened were estimated by applying a multiplier to the proportions screened among all insured women, as estimated from claims data. The ratio of proportion screened among uninsured to insured was estimated by region, using data from the Behavioral Risk Factor Surveillance System, averaged across years 2008–2020 (https://www.cdc.gov/brfss/index.html). This ratio was then multiplied by the annual proportions screened for insured females.

†††
https://surveillance.cancer.gov/joinpoint

§§§ Joinpoint models were selected using weighted Bayesian information criterion. Up to two joinpoints were allowed; at least two observations were required between joinpoints or from a joinpoint to either end. If joinpoints were identified, segment-specific trends (APC) were reported. Trends were considered to increase when AAPC or APC >0 or decrease when AAPC or APC <0 and if the empirical quantile 95% CI did not include 0; otherwise, trends were considered stable.

¶¶¶ Analyses were also performed with 2020 data included.

**** 45 C.F.R. part 46.102(l)(2), 21 C.F.R. part 56; 42 U.S.C. Sect. 241(d); 5 U.S.C. Sect. 552a; 44 U.S.C. Sect. 3501 et seq.

††††
https://www.cdc.gov/mmwr/preview/mmwrhtml/su6004a9.htm

§§§§
https://www.cdc.gov/hpv/vaccines/index.html

¶¶¶¶
https://www.cdc.gov/cervical-cancer/screening/
